# Structural characterization and partial properties of dextran produced by *Leuconostoc mesenteroides* RSG7 from pepino

**DOI:** 10.3389/fmicb.2023.1108120

**Published:** 2023-02-02

**Authors:** Binbin Wang, Xiaoling Sun, Min Xu, Fengyi Wang, Weizhong Liu, Baomei Wu

**Affiliations:** ^1^School of Life Sciences, Shanxi Normal University, Taiyuan, China; ^2^School of Chemical Engineering and Technology, Tianjin University, Tianjin, China

**Keywords:** *Leuconostoc mesenteroides* RSG7, dextran, pepino, structural characterization, physical–chemical properties

## Abstract

Exopolysaccharides (EPSs) produced by lactic acid bacteria possess various bioactivities and potential attractions for scientific exploration and commercial development. An EPS-producing bacterial strain, RSG7, was previously isolated from the pepino and identified as *Leuconostoc mesenteroides*. Based on the analyses of high-performance size exclusion chromatography, high-performance ion chromatography, Fourier transform infrared spectroscopy, nuclear magnetic resonance spectroscopy, and methylation, the RSG7 EPS was identified as a dextran with a molecular weight of 5.47 × 10^6^ Da and consisted of α-(1→6) glycosidic linkages as backbone and α-(1→2), α-(1→3), α-(1→4), and α-(1→6) glycosidic linkages as side chains. Scanning electron microscopy observed a honeycomb-like porous structure of RSG7 dextran, and this dextran formed aggregations with irregular hill-shaped lumps according to atomic force microscopy analysis. Physical–chemical investigations suggested that RSG7 dextran possessed excellent viscosity at high concentration, low temperature, and high pH; showed a superior emulsifying capacity of tested vegetable oils than that of hydrocarbons; and owned the maximal flocculating activity (10.74 ± 0.23) and flocculating rate (93.46 ± 0.07%) in the suspended solid of activated carbon. In addition, the dextran could coagulate sucrose-supplemented milk and implied potential probiotics *in vitro*. Together, these results collectively describe a valuable dextran with unique characteristics for exploitation in food applications.

## 1. Introduction

Lactic acid bacteria (LAB) are generally regarded as safe (GRAS) for human beings in their daily diet and could be commonly isolated from fermented foods, including wine, yogurt, milk, and kimchi (Kook et al., 2019); meanwhile, the LAB is also found in the meat and vegetable origin and even in fruits (Ruiz Rodríguez et al., 2019). These bacteria can produce abundant organic acids (mainly lactic acid) and metabolites. Exopolysaccharide (EPS) is one of the important and bioactive compounds of many LAB metabolites. EPS is produced by not only LAB but also by yeasts, algae, fungi, molds, plants, and animals, and microbial EPS especially from LAB has, however, drawn extensive attention according to their convenience of extraction and purification (Wang et al., 2019a,b). Based on the units of the monosaccharides, the EPS can be divided into homopolysaccharide (HoPS) and heteropolysaccharide (HePS); HoPS is composed of only one single type of monosaccharide, whereas HePS consists of two or more types of monosaccharides (Lynch et al., 2018). Both the HoPS and HePS have potential applications in food and dairy industries as gelling, stabilizing, emulsifying, thickening, water-banding, and viscosifying agents and in the field of the pharmaceutical industry as an antioxidant, immunomodulator, anticancer, anticoagulant, antiviral, and anti-cholesterol agents (Daba et al., 2021).

Homopolysaccharides, glucans, and fructans with glucose and fructose as monosaccharide units, respectively, are synthesized from sucrose by glucansucrases (Lynch et al., 2018). The glucans can be divided into α- and β-glucans due to the molecular configuration, and the former is more widely isolated in the LAB (Dubey and Jeevaratnam, 2015). According to the linkage types of glycosidic bonds in the polymer backbone, the α-glucans are subdivided into dextrans with α-(1→6) linkages, mutan with α-(1→3) linkages, reuterans with α-(1→4) linkages, and alternans with α-(1→3) and α-(1→6) linkages, which may own side-chain branches (Dubey and Jeevaratnam, 2015; Singh and Saini, 2017). For example, the dextran produced by *Apilactobacillus kunkeei* AK1 (former *Lactobacillus kunkeei* AK1) contained 4.78% α-(1→4) glycosidic linkage branches apart from the main chains of α-(1→6) linkages (Zheng et al., 2020; Yilmaz et al., 2022a). Several reports have suggested that the dextrans are usually produced by the LAB of the geuns *Lactobacillus*, *Leuconostoc*, *Pediococcus*, *Streptococcus*, and *Weissella* (Heinze et al., 2006; Kothari et al., 2015; Díaz-Montes, 2021). Among these LAB, *Leuconostoc* species are the primary producers of the dextran and rigorous proofs of the dextran-synthesizing activity were published in 1939 and 1941 (De Belder, 1993). Currently, *Leu. mesenteroides* NRRL B512 has been used widely and successfully to obtain dextran to satisfy practical needs in the industry and clinical area (Díaz-Montes, 2021). However, there is still a lack of high-quality functional *Leu. mesenteroides* to satisfy the requirements of large-scale industrial applications. Consequently, screening new *Leu. mesenteroides*, which could produce EPS with special morphology, excellent physical–chemical properties, and biological activities, from interesting materials still possess potential values in scientific research.

Pepino (*Solanum muricatum* Aiton) with a wonderful Chinese name “Ren Shen Guo” is a succulent, juicy, and sweet fruit and is considered a potential model of textural alteration during fruit ripening (Heyes et al., 1994; Pandey et al., 2021). Several studies suggested that *Leuconostoc* was the most abundant type of LAB distributed in most of the studied fruit samples (Trias et al., 2008; Fessard and Remize, 2019; Ruiz Rodríguez et al., 2019). However, there is little information relating to *Leu. mesenteroides* isolated from pepino, to say nothing of the EPS produced by this bacterium in the fruit. Therefore, based on our previous isolation of one strain, *Leu. mesenteroides* RSG7, from the pepino, the EPS produced by this strain was comprehensively studied. The molecular mass and monosaccharide composition were first determined by high-performance size exclusion chromatography (HPSEC) and high-performance ion chromatography (HPIC) according to standards, respectively. Subsequently, the structural characteristics of RSG7 EPS were separately analyzed by Fourier transform infrared (FT-IR) spectroscopy, nuclear magnetic resonance (NMR) spectroscopy, and methylation, and then, the morphological and topographical features were observed with scanning electron microscopy (SEM) and atomic force microscopy (AFM). Moreover, physical–chemical properties and partial biological activities were also investigated to evaluate its potential applications in food fermentation processing and industrial fields.

## 2. Materials and methods

### 2.1. Microorganism and medium preparation

*Leu. mesenteroides* RSG7 was isolated from pepino (Linfen, Shanxi, China) with an NCBI GenBank accession number: SUB12148640 *Leuconostoc* OP627195. *Lacticaseibacillus casei* ATCC393, *Lactobacillus acidophilus* NCFM, *Lactiplantibacillus plantarum* FS, *Streptococcus thermophilus*, *Bifidobacterium animalis* ATCC27673, and *Bifidobacterium longum* LTBL16 were kindly provided by professor Zhijiang Zhou from Tianjin University (Tianjin, China). MRS (De Man, Rogosa, Sharpe) medium contained 20 g of glucose, 10 g of beef extract, 10 g of tryptone, 5 g of anhydrous sodium acetate, 5 g of yeast extract, 2 g of ammonium citrate, 2 g of K_2_HPO_4_, 0.58 g of MgSO_4_⋅7H_2_O, 0.25 g of MnSO_4_⋅H_2_O, and 1 ml Tween 80 in 1 L distilled water (Zhou et al., 2018).

### 2.2. Extraction and purification of RSG7 EPS

The RSG7 EPS was extracted and purified according to our previous studies (Wang et al., 2019a,b). In brief, the RSG7 strain was first cultivated in liquid MRS medium containing 5% sucrose with a shaking speed of 110 rpm for 48 h at 30^°^C. Then, the bacteria cells were separated by centrifugation (8,000 × *g*, 60 min, 4^°^C), and the supernatant was treated with three volumes of precooled ethanol (95%) to precipitate the EPS overnight at 4^°^C. After centrifugation (8,000 × *g*, 60 min, 4^°^C), the precipitate was obtained and dissolved in 100 ml of distilled water; then, proteins were removed by adding an equal volume of 10% trichloroacetic acid (TCA) to the abovementioned sample. The supernatant was collected after centrifugation at 8,000 × *g* for 60 min and mixed with three volumes of precooled ethanol (95%) overnight at 4^°^C. Subsequently, the precipitate was pelleted by centrifugation (8,000 × *g*, 60 min, 4^°^C), dissolved in deionized water, and dialyzed (membrane cutoff 14,000 Da) to eliminate the small molecules or ions for 2 days at 4^°^C. The obtained crude RSG7 EPS solution was further purified by gel-filtration chromatography with a Sephadex G-100 column (1.6 × 50 cm, GE Healthcare, Fairfield, CT, USA) using deionized water at a flow rate of 0.2 ml/min and a detection wavelength of 220 nm. The purity of collections was confirmed by UV/visible Spectrophotometer (SPECCORD ^®^ 200 PLUS, Jena, Germany) over a wavelength range of 190–350 nm. Finally, the purified EPS was lyophilized for further studies.

### 2.3. Determination of RSG7 EPS molecular weight (Mw)

The Mw of RSG7 EPS was determined by HPSEC (LC-10A, Shimadzu, Kyoto, Japan) with a BRT105-104-102 tandem gel column (Φ8.0 × 300 mm, BoRui Saccharide, Yangzhou, China) and refractive index detector (RI-10A, Shimadzu, Kyoto, Japan). The RSG7 EPS was dissolved in distilled water with a final concentration of 5 mg/ml and filtered through a 0.22 μm membrane filter before injection. Then, 20 μl of aliquot was examined with a flow rate of 0.6 ml/min and column temperature of 40^°^C. The data were calculated with a calibration curve constructed by dextran standards (Mw = 1,152–667,800 Da, Yuanye and Sigma).

### 2.4. Monosaccharide composition analysis

The monosaccharide composition of RSG7 EPS was analyzed by HPIC (ICS5000, Thermo Fisher, Waltham, MA, USA). Purified RSG7 EPS (5 mg) was hydrolyzed with 2 ml of 3 mol/L trifluoroacetic acid (TFA) at 120^°^C for 3 h, and the residual TFA in the hydrolyzates was removed by nitrogen flow. Subsequently, the dried hydrolyzates were dissolved in 5 ml deionized water, and 50 μl solution with 950 μl deionized water was mixed and centrifuged at 12,000 rpm for 5 min; then, the supernatant was analyzed by HPIC using an electrochemical detector and CarboPacTM PA20 (Φ3.0 × 150 mm) column at a temperature of 30^°^C with a mobile phase consisting of deionized water (A), 15 mmol/L NaOH (B), 15 mmol/L NaOH, and 100 mmol/L NaOAc (C) at a flow rate of 0.3 ml/min. The monosaccharide composition of RSG7 EPS was determined by comparing it with standard sugars [fucose (Fuc), galactosamine hydrochloride (GalN), rhamnose (Rha), arabinose (Ara), glucosamine hydrochloride (GlcN), galactose (Gal), glucose (Glc), N-acetyl-D-glucosamine (GlcNAc), xylose (Xyl), mannose (Man), fructose (Fru), and ribose (Rib)] based on retention time (RT).

### 2.5. FT-IR spectroscopy analysis

The FT-IR spectrum of RSG7 EPS was analyzed by a TENSOR 27 spectrometer (Bruker, Karlsruhe, Germany). The purified EPS was mixed with dried KBr powder in a ratio of 1:100 and pressed into 1 mm pellets for FT-IR measurement within the scanning range of 500 and 4,000 cm^–1^.

### 2.6. NMR spectroscopy analysis

A total of 30 mg purified RSG EPS was dissolved in 99.9% D_2_O (Tenglong Weibo, Qingdao, China), and then, H/D exchange was conducted three times for NMR. The ^1^H NMR, ^13^C NMR, DEPT135 spectra, and two-dimensional spectra [^1^H-^1^H correlation spectroscopy (COSY), ^1^H-^13^C heteronuclear single quantum coherence (HSQC), and ^1^H-^13^C heteronuclear multiple bond correlation (HMBC)] were recorded using a Bruker 400 liquid NMR spectrometer (Bruker Biospin, Rheinstetten, Germany), and all data were analyzed using MestReNova software.

### 2.7. Methylation analysis

Methylation analysis of RSG7 EPS was carried out according to previous studies (Llamas-Arriba et al., 2019; Wangpaiboon et al., 2020). In brief, 2–3 mg RSG7 EPS was fully dissolved in 500 μl DMSO, where 1 mg NaOH powder was added. After incubation for 30 min, the samples were methylated with 50 μl CH_3_I for 1 h. Then, 1 ml of distilled water and 2 ml of CH_2_Cl_2_ were used to wash the methylated EPS (repeated for three times). The 100 μl of 2 mol/L TFA was added to the methylated samples for complete hydrolysis at 121^°^C for 90 min. Subsequently, methylated monosaccharides were reduced by adding 50 μl of 2 mol/L NaBD_4_ under an alkaline environment for 2.5 h. Then, acetylation was performed with 250 μl (CH_3_CO)_2_O for 2.5 h at 100^°^C. Finally, partially methylated alditol acetates were dissolved in 250 μl CH_2_Cl_2_ for GC-MS analysis (Agilent Technology 7890A-5977B, Palo Alto, CA, USA), which was equipped with a BPX70 column (SGE, Ringwood, Australia). The GC temperature program was isothermal at 140^°^C, followed by a 3^°^C/min gradient up to 230^°^C.

### 2.8. SEM and AFM analyses

To determine the surface morphology and microstructure of RSG7 dextran, purified dextran samples were placed on the sample stage of the instrument (Zeiss Merlin, Oberkochen, Germany) and gold-sputtered. The SEM images were observed by applying three magnification levels (0.5 ×, 1.0 ×, and 2.0 k ×) with an accelerating voltage of 5.0 KV.

Atomic force microscopy analysis was conducted with a scanning probe microscope (NTEGRA Spectra, NT-MDT, Moscow, Russia) in tapping mode. A total of 1 mg/ml purified dextran aqueous solution was continuously stirred for about 1 h in a sealed bottle under an N_2_ stream at 40^°^C. After cooling to room temperature, the mixture was diluted to the final concentration of 10 μg/ml. Then, a 5 μl diluted mixture was dropped on the mica sample carrier (Pelco 10 mm) which was then dried at room temperature. Topography observation and morphological parameters were performed by the instrument and NanoScope software, respectively.

### 2.9. Rheological analysis

The dynamic rheological manner of the RSG7 dextran was analyzed with a viscometer (NDJ-8S, Shanghai Lichen Bangxi Instrument Technology Co., Ltd., Shanghai, China). The lyophilized dextran was dissolved in distilled water with different concentrations (40, 50, 60, 70, and 80 mg/ml), and viscosities were subsequently measured at altered rotor speeds (0.3, 0.6, 1.5, 3, 6, 12, 30, and 60 rpm). The effect of temperatures (4, 25, 35, 45, and 55^°^C) and pH values (1.0, 4.0, 9.0, and 12.0) on the dextran (40 mg/mL) viscosities were also explored at the abovementioned rotor speeds. All measurements were conducted three times.

### 2.10. Emulsifying activity and stability

The percentage retention of the emulsion after incubation for 1 h (emulsification activity, %EA) and emulsification indexes after 24, 48, and 72 h (emulsion stability, %ES) were assayed according to the method described by Kanamarlapudi and Muddada (2017). For evaluation, RSG7 dextran was fully dissolved in distilled water at various concentrations (0.5, 1.0, and 1.5 mg/mL). Next, 1.5 ml of the dextran solution was added to an equal volume of hydrocarbons (kerosene, petrol, diesel oil, benzene, methylbenzene, dimethylbenzene, n-hexane, and cyclohexane) or vegetable oils (soybean oil, sunflower oil, peanut oil, and rapeseed oil), and the mixture was fully stirred in a vortex for 2 min. The emulsion indexes were determined as given below:

%EA or %ES = (height of emulsion layer/overall height of mixture) × 100%.

Subsequently, the emulsion formed after 24 h storage was observed using a stereoscopic microscope (ZEISS, Axio Zoom v16, Oberkochen, Germany) by placing 100 μl of the emulsion in a microscope slide.

### 2.11. Flocculation characteristics analysis

The flocculation activity and flocculating rate of RSG7 dextran were measured as described by Han et al. (2014), Krishnamurthy et al. (2020) with slight modifications. Generally, 1 ml of different diluted dextran solutions (10–500 mg/L), 1 ml of 1% CaCl_2_, and 8 ml of 5 g/L activated carbon suspension were mixed in a test tube. Such mixture was vortexed for 2 min and stood for 5 min. In total, 3 ml of supernatant was collected instantly from the upper layer and its absorbance was measured at 550 nm with a spectrophotometer. The flocculation activity and flocculating rate were estimated according to the following equations:

Flocculating activity = 1/A–1/B; Flocculating rate = (B–A)/B × 100%

where “A” and “B” are optical density values (550 nm) of RSG7 dextran samples and blank (double distilled water instead of EPS) samples, respectively. Flocculation characteristics of inorganic flocculant Al_2_(SO_4_)_3_ and commercial EPS Guar gum were also conducted by keeping other parameters constant. The flocculating activity was provided in bar graph form, and the flocculating rate was represented by the solid line.

### 2.12. Skimmed milk coagulation test

A total of 5% (v/v) *Leu. mesenteroides* RSG7 pre-cultivated in MRS medium was inoculated into 10% (w/v) sterile skimmed milk containing 0, 3, 6, 9, and 12% (w/v) sucrose. Solidification of the fermented milk was observed and photographed after incubation for 24 h at 30^°^C.

### 2.13. *In vitro* fermentation of RSG7 dextran with *Lactobacillus*, *Streptococcus*, and *Bifidobacterium*

*Lac. casei* ATCC393, *Lac. acidophilus* NCFM, *Lac. plantarum* FS, and *S. thermophilus* were cultivated in MRS medium, and *B. animalis* ATCC27673 and *B. longum* LTBL16 were cultured in MRS medium containing 0.05% L-cysteine hydrochloride at 37^°^C under anaerobic conditions according to the previous report (Pan et al., 2020). RSG7 dextran (1%, w/v) and inulin (1%, w/v), one representative oligosaccharide prebiotics were used as a substitute for glucose in the MRS medium, while the MRS medium without glucose was designated as the control group. The growth curves of *Lac. acidophilus* NCFM and *B. longum* LTBL16 were performed for 72 h, and the other four bacteria were performed for 36 h. The final biomass of selected bacteria was measured with a UV/visible Spectrophotometer (SPECCORD ^®^ 200 PLUS, Jena, Germany) at 600 nm. All the measurements were conducted three times.

### 2.14. Statistical analysis

The data were expressed as mean ± SD and analyzed using SPSS software. Significant differences were evaluated by one-way analysis of variance (ANOVA).

## 3. Results and discussion

### 3.1. Extraction and purification of RSG7 EPS

**FIGURE 1:**
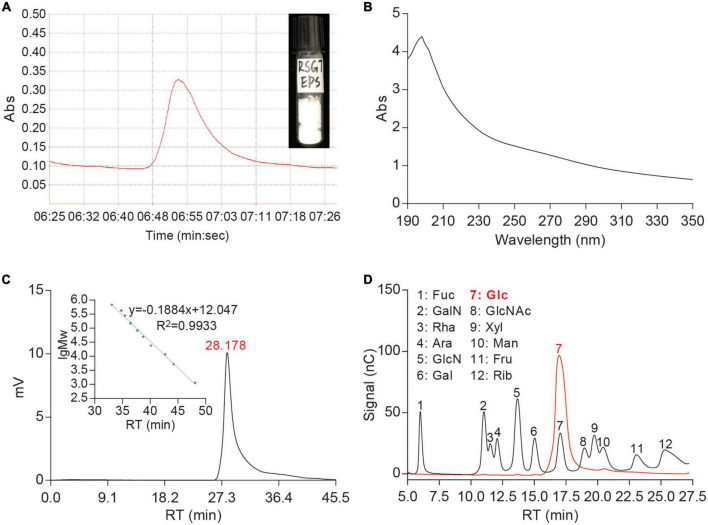
Profiles of *Leu. mesenteroides* RSG7 exopolysaccharide (EPS). **(A)** Sephadex G-100 chromatogram, **(B)** ultraviolet (UV) spectra, **(C)** molecular weight (Mw), and **(D)** monosaccharide composition.

The crude EPS was obtained from the fermentation broth of RSG7 strain by ethanol precipitation and TCA deproteinization. As shown in Figure 1A, this EPS was further purified by Sephadex G-100 gel filtration column chromatography. There was only one EPS peak on the elution curve, indicating a homogeneous sample. The purified fraction was collected and freeze-dried, white and fluffy solid EPS was obtained, and its water solution showed characteristic absorption peak between 190 and 210 nm and no pronounced absorption peaks at 260 and 280 nm with UV/vis spectroscopy analysis, indicating the purified RSG7 EPS did not contain nucleic acid and protein (Figure 1B).

### 3.2. Molecular mass and monosaccharide composition analyses of RSG7 EPS

The RT is significantly correlated with the molecular weight of EPS (Figure 1C). According to the HPSEC analysis, the Mw of RSG7 EPS corresponding to an RT of 28.178 min was determined to be 5.47 × 10^6^ Da (Figure 1C). A previous report suggested that the Mws of HePS produced by LAB ranges from 10^4^ to 10^6^ Da while that of HoPS is greater than 10^6^ Da (Lynch et al., 2018). Accordingly, the Mw of RSG7 EPS was consistent with that of HoPS. EPSs from different *Leu. mesenteroides* possess multiple Mws. Some strains have been reported to produce EPSs with lower Mws ranging between 1.0 × 10^4^ and 1.8 × 10^6^ Da (Park et al., 2013; Matsuzaki et al., 2015; Abid et al., 2018; Ziadi et al., 2018; Kook et al., 2019; Li et al., 2020; Zhang et al., 2021). Some strains, such as *Leu. mesenteroides* KIBGE-IB22, KIBGE-IB22M20, BI-20, and TDS2-19, in turn, were shown to produce higher Mws EPSs ranging between 1.5 × 10^7^ and 1.0 × 10^8^ Da (Siddiqui et al., 2014; Du et al., 2017; Yilmaz et al., 2022b). These comparisons implied that the Mw of RSG7 EPS belonged to the relatively large one among the known EPSs isolated from the *Leu. mesenteroides*. Several studies suggested that Mw of EPS played important roles in its biological activities and solution viscosity (Yang et al., 2022; Zhu et al., 2022). The higher Mw might endow RSG7 EPS with excellent viscosity during milk fermentation and solidification.

The HPIC was performed to examine the monosaccharide composition of RSG7 EPS after hydrolysis based on the standards. The results are presented in Figure 1D; compared with standards, only one peak appeared in the sample of RSG7 EPS, which was assigned to the Glc, indicating this EPS was a glucan.

### 3.3. FT-IR spectra of RSG7 EPS

**FIGURE 2:**
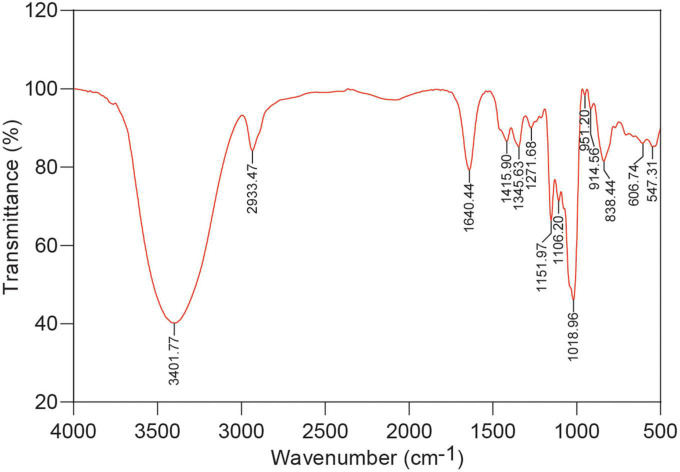
Fourier transform infrared (FT-IR) spectra of exopolysaccharide (EPS) obtained from *Leu. mesenteroides* RSG7.

Fourier transform infrared spectroscopy has been widely used to identify the structural and functional groups of EPS. Numerous peaks were observed in the FT-IR spectrum of purified RSG7 EPS from 4,000 to 500 cm^–1^ (Figure 2). The wavenumber from 1,200 to 800 cm^–1^ is the fingerprint region and is mainly caused by the vibrational state of the monomer glucose unit in the RSG7 EPS (Kizil et al., 2002), which only consisted of glucose according to HPIC analysis (Figure 1D). A previous study also indicated the presence of α-(1→6) glycosidic linkage based on the peak of 1,018.96 cm^–1^ (Miao et al., 2014). The band at 914.56 cm^–1^ was attributed to the pyranose ring from the glucosyl residue (Shao et al., 2014; Zhou et al., 2018) and 838.44 cm^–1^ was the characteristic of α-Glc (Zheng et al., 2019; Velichko et al., 2020), indicating that RSG7 EPS contains typical of α- anomers of Glc. The peaks at the region of 500–800 cm^–1^ further implied the presence of α-glycosidic linkages (Naibaho et al., 2022).

### 3.4. NMR spectroscopy

**FIGURE 3:**
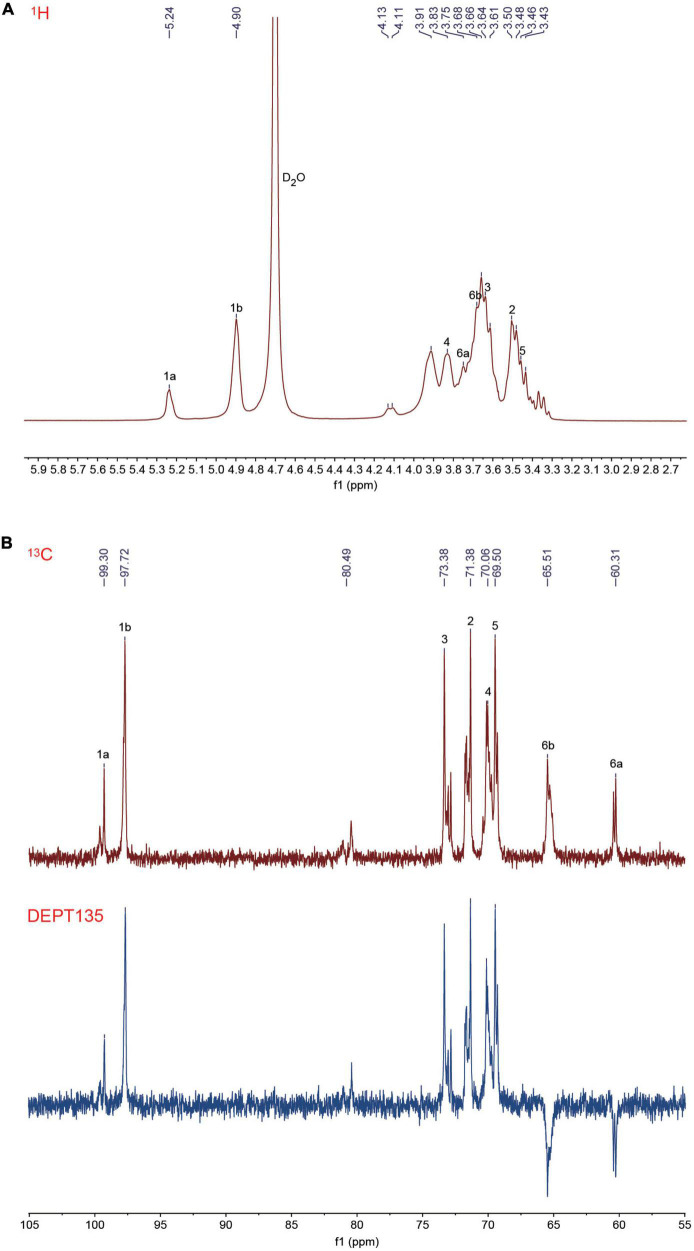
One-dimensional nuclear magnetic resonance (NMR) spectra of *Leu. mesenteroides* RSG7 exopolysaccharide (EPS). **(A)** ^1^H and **(B)** ^13^C and DEPT135.

The glycosidic bond configuration and molecular structure of the RSG7 EPS were further defined with one-dimensional (^1^H, ^13^C, and DEPT135) and two-dimensional (^1^H-^1^H COSY, ^1^H-^13^C HSQC, and ^1^H-^13^C HMBC) NMR spectroscopy. According to the ^1^H NMR of RSG7 EPS (Figure 3A), the anomeric region and ring proton region were detected at the signals of δ 4.5–5.5 and δ 3.1–4.5 ppm, respectively (Kavitake et al., 2016). The anomeric proton signal at 4.90 ppm was assigned to α-(1→6) glycosidic linkage, which was consistent with the FT-IR result at the absorption peak of 1,018.96 cm^–1^ (Figure 2). While the anomeric proton signal at 5.24 ppm was attributed to the presence of α-(1→3) glycosidic linkage in the RSG7 EPS (Li et al., 2022). However, the extensive overlapping of the peaks in the ring proton region due to the shielding effects of hydroxyl groups in the EPS resulted in difficulties in the attribution of each signal, and a two-dimensional NMR should be carried out to distinguish the signals. In the ^13^C NMR spectrum (Figure 3B), the anomeric carbon region was presented at δ 95–110 ppm and the ring carbon region at δ 50–85 ppm (Wang et al., 2019b). The typical peaks at 97.72 and 99.30 ppm corresponded to signals in the anomeric region of ^1^H NMR. According to these results, the anomeric carbon signals at 97.72 and 99.30 ppm and the anomeric region at 4.90 and 5.24 ppm were assigned to C1 (Figure 3B) and H1 (Figure 3A) in glucosyl residues, respectively. Meanwhile, peaks in the 77–85 ppm illustrated the presence of branched linkage in the RSG7 EPS (Kavitake et al., 2016). In addition, carbon resonances at 73.38, 71.38, 70.06, 69.50, 65.51, and 60.31 ppm were attributed to the C2–C6 in the glucosyl residues. Further analysis based on ^13^C NMR and DEPT135 spectrum suggested that peaks of 65.51 and 60.31 ppm were both assigned to the C6 in glucosyl residues, and the C6 of the former peak involved in the formation of glycosidic bonds, while the latter was unsubstituted in the RSG7 EPS.

**FIGURE 4:**
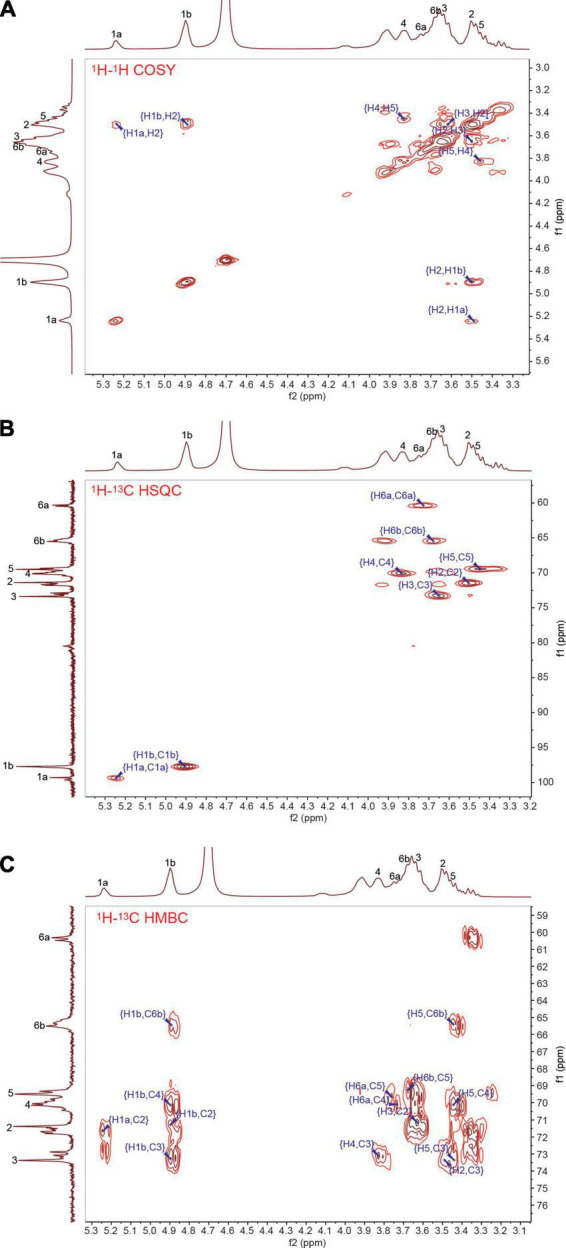
Two-dimensional nuclear magnetic resonance (NMR) spectra of *Leu. mesenteroides* RSG7 exopolysaccharide (EPS). **(A)** ^1^H-^1^H COSY, **(B)** ^1^H-^13^C HSQC, and **(C)** ^1^H-^13^C HMBC.

The ^1^H-^1^H COSY spectrum suggested that 3.50 and 3.64 ppm were separately assigned to the H2 and H3 in the glucosyl residues in accordance with the correlations between H1a, H1b, and the two signals (Figure 4A). The further analysis combined with the ^1^H-^13^C HSQC spectrum (Figure 4B) indicated that the chemical shift of signals at 71.38, 73.38, 3.72, and 3.67 ppm corresponded to C2, C3, H6a, and H6b, respectively. Meanwhile, the cross signals at 5.24/99.30 ppm (H1a/C1a) and 4.90/97.72 ppm (H1b/C1b) were observed. Furthermore, the intensity of cross-peaks at 3.45 and 65.51 ppm (C6b) was significantly stronger than that at 3.84 and 65.51 (C6b), and the signal of 3.84 ppm showed a visible cross signal with 73.38 ppm in the ^1^H-^13^C HMBC spectrum (Figure 4C); these results showed that the signals of 3.45/69.50 and 3.84/70.06 ppm were separately assigned to the H5/C5 and H4/C4. In addition, the signal of 4.90 ppm (H1b) showed strong cross-peaks with 71.38 ppm (C2), 73.38 ppm (C3), 70.06 ppm (C4), and 65.51 ppm (C6b) in the ^1^H-^13^C HMBC spectrum, implying the presence of α-(1→2), α-(1→3), α-(1→4), and α-(1→6) glycosidic linkages.

### 3.5. Methylation analysis of RSG7 EPS

**FIGURE 5:**
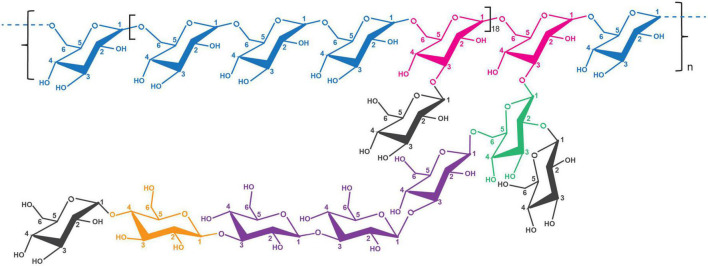
Proposed chemical structure of RSG7 exopolysaccharide (EPS).

Methylation analysis was carried out to deduce the glycosidic bonds of the monosaccharides in the RSG7 EPS. The linkage patterns of this EPS are summarized in Table 1, and GC-MS identifications of detail peaks are presented in the Supplementary Figure 1. The results suggested that RSG7 EPS had six derivatives, including 1,5-di-O-acetyl-2,3,4,6-tetra-O-methyl glucitol, 1,3,5-tri-O-acetyl-2,4,6-tri-O-methyl glucitol, 1,5,6-tri-O-acetyl-2,3,4-tri-O-methyl glucitol, 1,4,5-tri-O-acetyl-2,3,6-tri-O-methyl glucitol, 1,3,5,6-tetra-O-acetyl-2,4-di-O-methyl glucitol, and 1,2,5,6-tetra-O-acetyl-3,4-di-O-methyl glucitol at a molar ratio of 27.732: 2.792: 49.813: 0.690: 17.616: 1.357, which revealed the presence of a tail of (1→)-linked-D-Glcp, (1→3)-linked-D-Glcp, (1→6)-linked-D-Glcp, (1→4)-linked-D-Glcp, →3,6)-Glc(p)-(1→ and →2,6)-Glc(p)-(1→. Combined with monosaccharide composition, FT-IR, and NMR analyses, one possible molecular structure of the RSG7 EPS is shown in Figure 5, and this EPS was determined to be a dextran with α-(1→6) glycosidic linkages in the main chain and α-(1→2), α-(1→3), α-(1→4), α-(1→6) glycosidic linkages in branch point.

**TABLE 1:** Methylation analysis of RSG7 exopolysaccharide (EPS).

Deduced linkage	Methylation products	Molar ratios (%)^1^	Time (min)	Major mass fragments (m/z)
T-Glcp-(1→	1,5-di-O-acetyl-2,3,4,6-tetra-O-methyl glucitol	27.732	8.377	71, 87, 102, 129, 145, 162, 205
→3)-Glcp-(1→	1,3,5-tri-O-acetyl-2,4,6-tri-O-methyl glucitol	2.792	11.495	71, 87, 101, 118, 129, 161, 234
→6)-Glcp-(1→	1,5,6-tri-O-acetyl-2,3,4-tri-O-methyl glucitol	49.813	13.078	71, 87, 102, 118, 129, 162, 173, 189, 233
→4)-Glcp-(1→	1,4,5-tri-O-acetyl-2,3,6-tri-O-methyl glucitol	0.690	13.358	60, 71, 87, 102, 118, 129, 162, 233
→3,6)-Glc(p)-(1→	1,3,5,6-tetra-O-acetyl-2,4-di-O-methyl glucitol	17.616	17.011	59, 74, 87, 101, 118, 129, 139, 160, 189, 234
→2,6)-Glc(p)-(1→	1,2,5,6-tetra-O-acetyl-3,4-di-O-methyl glucitol	1.357	17.632	60, 87, 99, 118, 130, 145, 189

^1^Relative percentage of all derivatives based on the peak area.

### 3.6. SEM and AFM analyses

**FIGURE 6:**
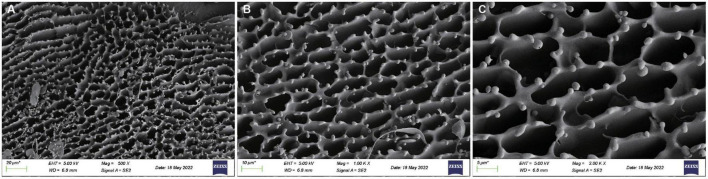
Scanning electron microscopy (SEM) images of *Leu. mesenteroides* RSG7 dextran. **(A)** 0.5 k ×, **(B)** 1.0 k ×, and **(C)** 2.0 k ×.

Scanning electron microscopy is a powerful imaging tool to illustrate and characterize the surface morphology and texture of polysaccharide biopolymers. The images with different magnifications (0.5 ×, 1.0 × and 2.0 k ×) of RSG7 dextran are presented in Figure 6. RSG7 dextran showed a honeycomb-like porous structure with spike-like expansions around the pores, implying that it might have a good water-holding capacity, which was crucial for improving the physical–chemical properties of fermented foods. According to previous reports, different EPSs from *Leu. mesenteroides* were isolated with similar methods and characterized by SEM, including BI-20 EPS showing fibrous and branched structure (Yilmaz et al., 2022b), EPSs of N5 and N7 showing separately porous and tight structures (Ma’unatin et al., 2022), S81 EPS possessing a sheet-like compact structure (Taylan et al., 2019), and XR1 EPS exhibiting lotus leaf shape and compact structure (Wang et al., 2021). Consequently, the honeycomb-like porous structure of RSG7 dextran has been rarely reported, which indicated the unique feature.

**FIGURE 7:**
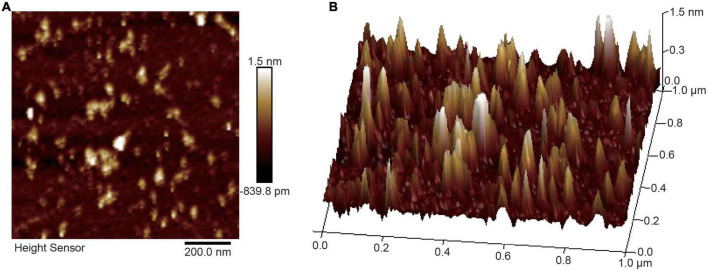
Atomic force microscopy (AFM) images of *Leu. mesenteroides* RSG7 dextran. **(A)** Planar. **(B)** Cubic.

The microstructure information of RSG7 dextran was analyzed by AFM, and the planar and cubic images are shown in Figure 7. The RSG7 dextran chain formed various sizes of aggregations. Microstructural images exhibited irregular hill-shaped lumps, EPS from *Leu. mesenteroides* S81 appeared spheroidal or ellipsoidal structural features (Taylan et al., 2019), and EPS from *Leu. mesenteroides* XR1 was granular with uniform distribution (Wang et al., 2022). These results fully proved that the microstructure of EPSs was strain-specific, even though in the same genus. Moreover, the peak height of RSG7 dextran aggregates varied between 0.30 and 3.25 nm, which was higher than the diameter of a single polysaccharide chain (0.1–1 nm) (Liu et al., 2020), suggesting that RSG7 dextran formed a cohesive structure due to the inter- or intra-molecular interaction behavior. These morphological characteristics considerably affect the biological activities and functional properties of RSG7 dextran (Hu et al., 2020).

### 3.7. Rheological properties of RSG7 dextran

**FIGURE 8:**
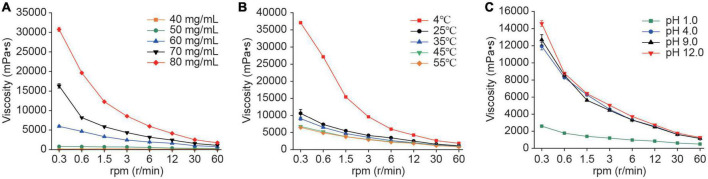
Rheological behavior of RSG7 dextran at different conditions. **(A)** Dextran concentrations within the range of 40–80 mg/mL, **(B)** temperatures of 4, 25, 35, 45, and 55^°^C, and **(C)** pH values of 1.0, 4.0, 9.0, and 12.0. Data represent the mean ± SD of three determinations.

The EPS viscosity was strongly correlated with polymer structure and Mw, which were affected by several factors such as concentration, temperature, and pH (Wang et al., 2019b). The alteration of RSG7 dextran’s apparent viscosity with three factors is illustrated in Figure 8. The results showed that the apparent viscosity of RSG7 dextran exhibited good pseudoplastic and non-Newtonian fluid behavior under the same concentration, temperature, and pH with the increase in rotor speed.

Further analysis showed that the apparent viscosity increased with the increase in RSG7 dextran concentrations at the same rotor speed, such as 0.3 rpm, and the apparent viscosity increased from 150 ± 15 to 30735 ± 658 mPa⋅s along with the concentration increase from 40 to 80 mg/L (Figure 8A). Similar trends were also observed in EPSs produced by *Weissella confusa* XG-3 (Zhao et al., 2020), *Latilactobacillus sakei* L3 (former *Lactobacillus sakei* L3; Wang et al., 2019b), and *Leu. mesenteroides* strain XR1 (Wang et al., 2021).

Meanwhile, the apparent viscosity decreased with the increase in temperatures (Figure 8B), and the trend at the same rotor speed was 4>25>35>45>5^°^C, which might be attributed to molecular motion that high temperature could accelerate molecular thermal movement to increase the intermolecular distance and weaken the interaction, causing the decline of apparent viscosity (Guo et al., 2022), and this result was similar to the previous reports for the EPSs from *Lac. helveticus* MB2-1 (Xiao et al., 2020) and *Lat. sakei* L3 (Wang et al., 2019b). These results indicated that RSG7 EPS might be applied commercially in food fermentation at an appropriate temperature.

In addition, varied microbial EPSs exhibited different apparent viscosity characteristics at altered pHs. EPS from *Vibrio alginolyticus* showed an increasing trend of apparent viscosity with pH increasing from 5.0 to 10.0 (Muralidharan and Jayachandran, 2003), while several LAB EPSs owned high viscosities at low pHs (Zhou et al., 2018; Wang et al., 2019b). Figure 8C suggests that the RSG7 dextran possessed high apparent viscosity from pH 4.0 to 12.0, which envisaged its potential use as a bio-thickener or fermented products stabilizer.

### 3.8. Emulsification properties

**FIGURE 9:**
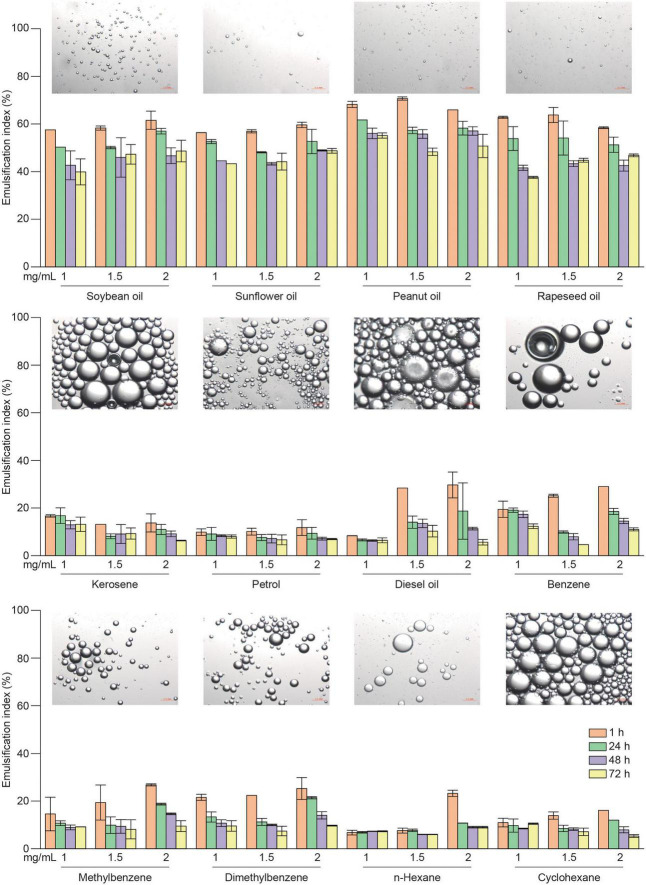
Emulsification properties of *Leu. mesenteroides* RSG7 dextran in different concentrations with vegetable oils and hydrocarbons. Data represent the mean ± SD of three determinations.

Emulsification and rheology are usually important indicators for judging the quality of dairy products and soy foods. The results given earlier showed that RSG7 dextran exhibited good pseudoplastic and non-Newtonian fluid behavior (Figure 8), and it could improve the textural properties of dairy products. To further study the emulsification index of RSG7 dextran, we prepared the emulsions with three concentrations of dextran and different vegetable oils/hydrocarbons. Figure 9 shows that the majority of the emulsion indices increased with an increasing dextran concentration, and RSG7 dextran exhibited a superior emulsifying capacity of tested vegetable oils than that of hydrocarbons. Dextran with all vegetable oils used in this study showed emulsion indices ranging from 37.59 ± 0.43 to 70.69 ± 0.67% and a gradual decrease toward 24, 48, and 72 h. The emulsion of RSG7 dextran with peanut oil exhibited the highest activity and stability, which were 70.69 ± 0.67, 61.71 ± 0.30, 57.04 ± 1.76, and 55.16 ± 1.10% at EA, ES_24_, ES_48_, and ES_72_ indices, respectively. Previous studies demonstrated that an efficient emulsifier should maintain at least 50% of the original emulsion volume until 24 h after its formation (Riaz Rajoka et al., 2018), and we found that RSG7 dextran possessed excellent emulsion stabilizing capacity for all tested vegetable oils, as shown by the ES_24_/EA indices of emulsions with soybean oil (92.6%), sunflower oil (88.3%), peanut oil (88.2%), and rapeseed oil (87.7%), respectively. The promising emulsification properties of RSG7 dextran might be used as a food emulsifier, especially in peanut oil.

Photomicrographs of different emulsions showed that large droplets were observed in the emulsions of dextran with hydrocarbons, whereas the emulsions of dextran with vegetable oils showed small and densely distributed droplets, which had higher emulsification indices. This emulsion droplet pattern was similar to that of *Nostoc flagelliforme* (Han et al., 2014) and *Bacillus amyloliquefaciens* LPL061 EPSs (Han et al., 2015). The emulsion droplet size is considered to be an important parameter of emulsions stability, and previous studies suggested that small densely distributed droplets could result in more stable emulsions (Han et al., 2015) that might explain the differences in emulsification properties between dextran with vegetable oils and hydrocarbons.

### 3.9. Flocculation characteristics

**FIGURE 10:**
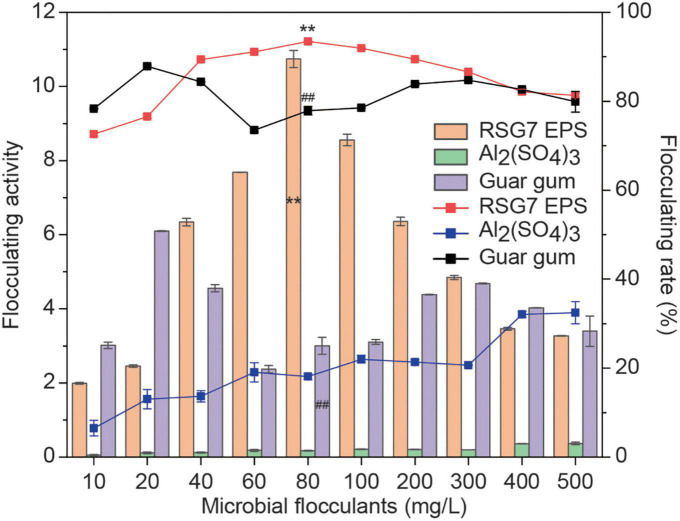
Flocculation activity and flocculation rate (%) of different microbial flocculants at a concentration range of 10–500 mg/L. Data represent the mean ± SD of three determinations. *p* < 0.01 is indicated by ** for RSG7 dextran versus Al_2_SO_4_, and *p* < 0.01 is indicated by ^##^ for Guar gum versus Al_2_SO_4_.

Microbial flocculants have been studied into three categories including inorganic flocculants [e.g., Al_2_(SO_4_)_3_], organic flocculants (e.g., polyacrylamide), and bioflocculants. Bioflocculants have become of particular interest because of their harmlessness, biodegradability, and excellent flocculation activity, which could be widely used in pollutant treatment, food fermentation processing, and mineral industries. EPS has been found to have flocculating property, some of which make them suitable candidates for alternative flocculants. Thus, in this present work, the flocculation characteristics of RSG7 dextran were tested with activated carbon as the suspended solid. Al_2_(SO_4_)_3_ and the commercial bioflocculants Guar gum were used for comparison. As shown in Figure 10, gradual increases in both the flocculating activity and flocculating rate were observed with an increasing RSG7 dextran concentration (10–80 mg/L), and thereafter, the flocculating activity and rate were reduced with the EPS dose increase. RSG7 dextran had the maximal flocculating activity (10.74 ± 0.23) and flocculating rate (93.46 ± 0.07%), followed by Guar gum (highest flocculating activity of 6.10 ± 0.01 and flocculating rate of 87.87 ± 0.25%) and Al_2_(SO_4_)_3_ (highest flocculating activity of 0.37 ± 0.04 and flocculating rate of 32.46 ± 2.50%). Several researchers found that bridging might be one of the flocculation mechanisms (Yim et al., 2007; Han et al., 2014), and Bezawada et al. (2013), Busi et al. (2017) reported that functional group and concentration of EPS played an essential role in flocculation characteristics. The appropriate concentration of EPS could promote the formation of bridges; however, with the increase in EPS concentration, the system of the suspension is re-steered due to electrostatic repulsion, and the flocculation effect decreases. The flocculating study of RSG7 dextran suggested its potential use as an alternative bioflocculant for several applications.

### 3.10. Skimmed milk solidification analysis

**FIGURE 11:**
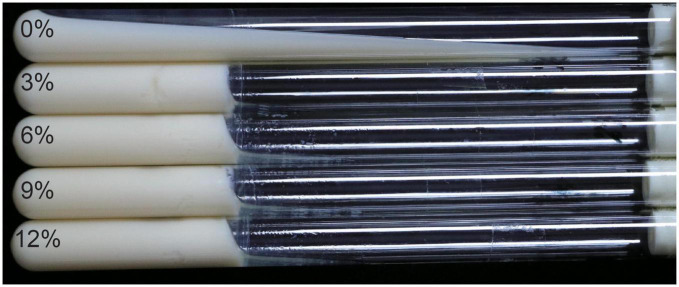
Effect of *Leu. mesenteroides* RSG7 dextran on the solidification of sucrose-supplemented skim milk. A total of 0, 3, 6, 9, and 12% represent the concentrations of sucrose.

As shown in Figure 11, milk solidification was observed by the addition of sucrose after 24 h incubation under the designed sucrose concentrations (0, 3, 6, 9, and 12%), compared with the control group with sucrose-free milk (0%). According to our abovementioned results, the *Leu. mesenteroides* RSG7 could synthesize dextran, which possessed excellent viscosity, in the presence of sucrose. This might be attributed to glucansucrases functioning in the synthesis of glucans in this strain (Bejar et al., 2013; Lynch et al., 2018). Meanwhile, a previous study suggested that the EPS molecule was able to interact with milk proteins (Ayala-Hernandez et al., 2008). Consequently, the milk solidification might be attributed to the physical–chemical properties of the RSG7 dextran and the interactions between dextran and dairy proteins. Further comparison indicated that the RSG7 dextran showed more excellent solidifying ability than EPS produced by *Levilactobacillus brevis* HDE-9 (Du et al., 2022) under the same sucrose (3%), skimmed milk concentration (10%), bacteria content (5%), and incubation time (24 h). Therefore, RSG7 dextran could be used as a safe food additive to improve the textural properties of sucrose-supplemented dairy products.

### 3.11. Growth of probiotics in the RSG7 dextran-containing medium

**FIGURE 12:**
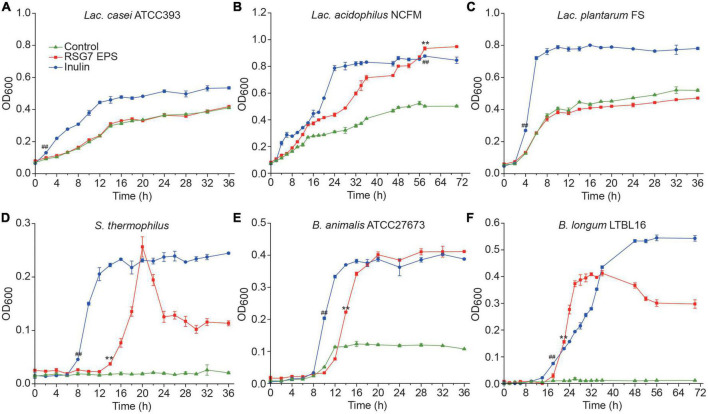
Growth profiles of *Lactobacillus*, *Streptococcus*, and *Bifidobacterium* on glucose-free MRS added with different carbon sources at 37^°^C under anaerobic treatments. **(A)** *Lac. casei* ATCC 393, **(B)** *Lac. acidophilus* NCFM, **(C)** *Lac. plantarum* FS, **(D)** *S. thermophilus*, **(E)** *B. animalis* ATCC 27673, and **(F)** *B. longum* LTBL16. Each point represents mean ± SD of three determinations. *p* < 0.01 is indicated by ** for RSG7 dextran versus control, and *p* < 0.01 is indicated by ^##^ for inulin versus control.

The RSG7 dextran was used to cultivate the probiotics including *Lactobacillus* (Figures 12A–C), *Streptococcus* (Figure 12D), and *Bifidobacterium* (Figures 12E, F). The OD_600_ values of medium inoculating *Lac. casei* ATCC393 with RSG7 dextran showed no difference with the control group; meanwhile, both of *Lac. casei* ATCC393 and *Lac. plantarum* FS with RSG7 dextran showed lower growth than that with inulin, which might attribute to the fact that the oligosaccharide was more conducive to the growth of LAB during the bacteria growth period. However, the bacterial growth of *Lac. acidophilus* NCFM in RSG7 dextran-containing medium reached a plateau at a higher OD value than that observed with the inulin and control group. Moreover, RSG7 dextran promoted the growth of *S. thermophilus*, *B. animalis* ATCC27673, and *B. longum* LTBL16 significantly in comparison with the control group. The logarithmic phase of *S. thermophilus* was delayed for 6 h during incubating with the RSG7 dextran in comparison with that in inulin, and the OD_600_ value achieved the highest point at 20 h and then decreased surprisingly to a stable tread. The optical density of *B. animalis* ATCC27673 and *B. longum* LTBL16 was separately delayed and stimulated to reach the stationary phase compared with that in inulin, which indicated that RSG7 dextran was associated with the two bacteria growth. These results differed from the previous study which investigated the relationships between XG5 EPS and several probiotics (Pan et al., 2020), implying different EPSs showed varied effects on the growth characteristics of the same probiotic.

## 4. Conclusion

In this study, a dextran produced by *Leu. mesenteroides* RSG7 isolated from pepino was investigated and characterized. The dextran was identified as HoPS and composed of Glc with an Mw of 5.47 × 10^6^ Da. The molecular structure analysis suggested RSG7 dextran contained mainly α-(1→6) glycosidic linkages as the backbone with complex glycosidic linkages in the branches, which endowed it with honeycomb-like porous structure and irregular hill-shaped lumps. Meanwhile, this dextran owned excellent rheological, emulsifying, and flocculating properties and allowed to solidify skimmed milk. Furthermore, RSG7 dextran showed different proliferation properties of *Lactobacillus*, *Streptococcus*, and *Bifidobacterium in vitro*. This study provides a basis for the potential application of the RSG7 dextran in food and industrial exploitation. However, further studies are required to determine the possible mechanisms of several excellent physical–chemical properties and focus on whether the RSG7 dextran plays roles in antimicrobial and immunomodulatory activities to adequately develop and utilize this dextran.

## Data availability statement

The datasets presented in this study can be found in online repositories. The names of the repository/repositories and accession number(s) can be found in the article/Supplementary material.

## Author contributions

BW and WL: conceptualization and writing—reviewing and editing. BW: methodology, data curation, formal analysis, writing—original draft preparation, and funding acquisition. XS, MX, and FW: validation, investigation, and visualization. All authors contributed to the article and approved the submitted version.

## Supplementary material

Supplementary Figure 1 Mass spectra for methylation analysis of RSG7 EPS. **(A)** T-Glcp-(1→; **(B)** →3)-Glcp-(1→; **(C)** →6)-Glcp-(1→; **(D)** →4)-Glcp-(1→; **(E)** →3,6)-Glcp-(1→; **(F)** →2,6)-Glc(p)-(1→.
