# Supplementary material for: Structural characterization and partial properties of dextran produced by Leuconostoc mesenteroides RSG7 from pepino
Source: Front Microbiol. 2023 Feb 2;14:1108120. doi: 10.3389/fmicb.2023.1108120 (PMC9933128; doi:10.3389/fmicb.2023.1108120)
Supplement: Supplementary Figure 1 — Mass spectra for methylation analysis of RSG7 EPS. (A) T-Glcp-(1→; (B) →3)-Glcp-(1→; (C) →6)-Glcp-(1→; (D) →4)-Glcp-(1→; (E) →3,6)-Glcp-(1→; (F) →2,6)-Glc(p)-(1→. [file Data_Sheet_1.docx]

Supplementary Material

Structural characterization and partial properties of dextran produced by *Leuconostoc mesenteroides* RSG7 from pepino

Binbin Wang^1†^, Xiaoling Sun^1†^, Min Xu^2^, Fengyi Wang^1^, Weizhong Liu^1*^ and Baomei Wu^1*^

^*^**Correspondence:**Weizhong Liu
liuwzh@sxnu.edu.cn

Baomei Wu
wubaomei@sxnu.edu.cn

^†^These authors contributed equally to this work

# Supplementary Figure


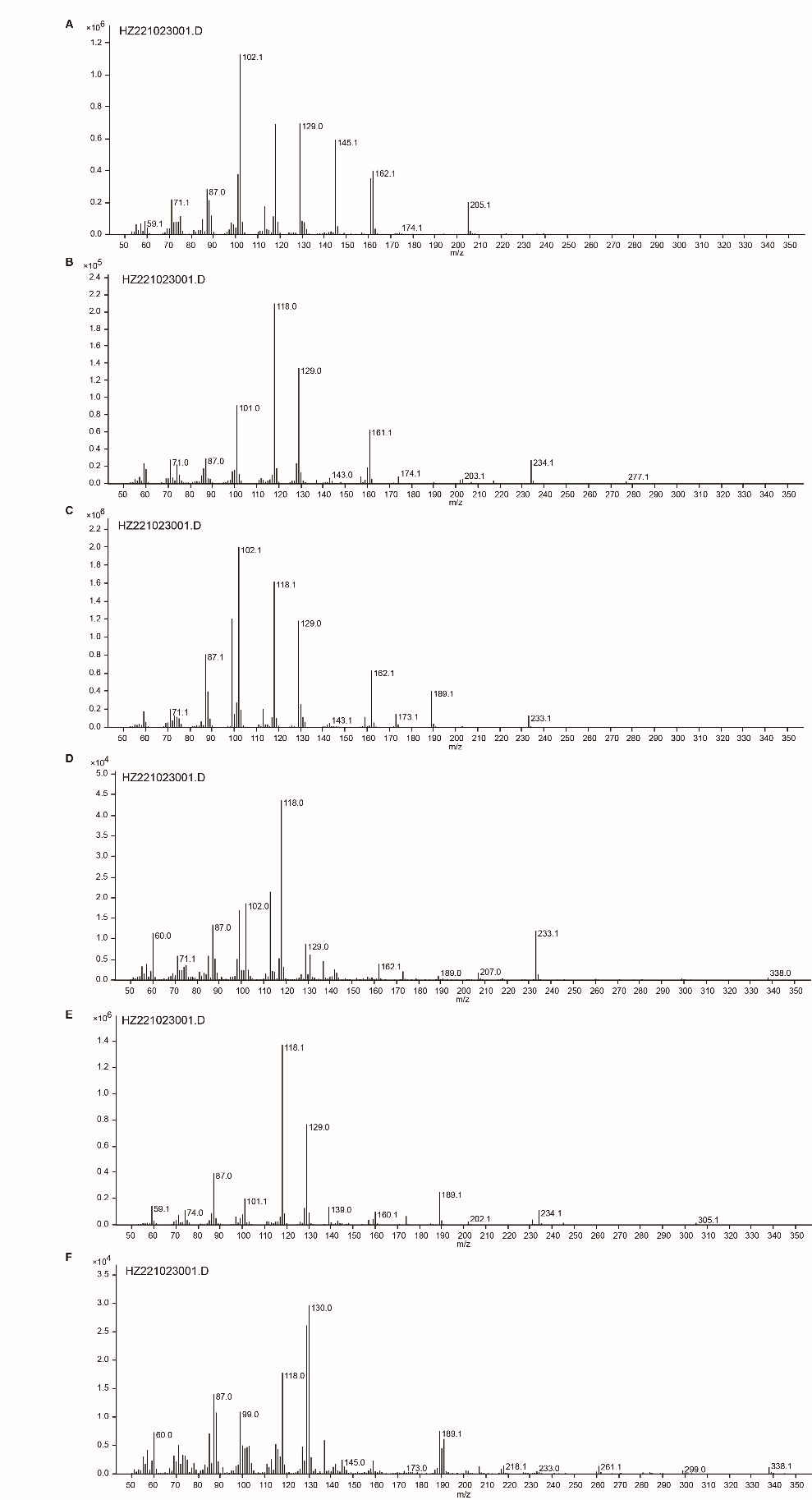


**Supplementary Figure 1.** Mass spectra for methylation analysis of RSG7 EPS. **(A)** T-Glcp-(1→, **(B)** →3)-Glcp-(1→, **(C)** →6)-Glcp-(1→, **(D)** →4)-Glcp-(1→, **(E)** →3,6)-Glcp-(1→, **(F)** →2,6)-Glc(p)-(1→.
